# The loading effect of Pt clusters on Pt/graphene nano sheets catalysts

**DOI:** 10.1038/s41598-020-80472-1

**Published:** 2021-01-28

**Authors:** Rikson Siburian, Ab Malik Marwan Ali, Kerista Sebayang, Minto Supeno, Kerista Tarigan, Crystina Simanjuntak, Sri Pratiwi Aritonang, Fajar Hutagalung

**Affiliations:** 1 Department of Chemistry, Faculty of Mathematics and Natural Sciences, Universitas Sumatera Utara, Padang Bulan, Medan, 20155 Indonesia; 2 Carbon Research Center, Universitas Sumatera Utara, Padang Bulan, Medan, 20155 Indonesia; 3 Faculty of Applied Sciences, Universiti Teknologi MARA, 40450 Shah Alam, Selangor Malaysia; 4 Department of Physics, Faculty of Mathematics and Natural Sciences, Universitas Sumatera Utara, Medan, 20155 Indonesia

**Keywords:** Chemistry, Materials science, Nanoscience and technology

## Abstract

In this paper, we report about chemically interaction between Pt Subnano-Clusters on Graphene Nano Sheets (GNS). The aim of this research is to clarify the size effect of Pt clusters on Pt 1–7 wt.%/GNS. This research is an experimental laboratory research. GNS was synthesized by using modified Hummer’s method and 1–7 wt.% Pt/GNS were prepared with impregnation method. Then, they were analyzed with TG/DTA, XRD, TEM and XPS, respectively. The results show that Pt clusters are well deposited on GNS (TG/DTA and TEM data). Those data also are consistent with XRD data. The weak and broad peaks appear at 2θ = 39°, indicating Pt metal exists on GNS. The state of Pt is confirmed by using XPS. The appearance of Pt 4f. peaks proves that Pt metal is chemical interaction on GNS. The size of Pt clusters may affect the chemically properties of Pt/GNS catalysts.

## Introduction

Carbon has been recognized as efficient metal-free electrocatalysts for ORR and OER in fuel cells and batteries, such as 2D graphene and 3D graphite^1^. Previously, we found that Pt Subnano clusters were well deposited on Graphene Nano Sheets (GNS). They also have the outstanding properties in term of catalytic activity and chemically interaction properties^2–8^. Fortunately, GNS also may be synthesized with large scale and facile method^9^ and it shows the remarkable properties as a support material^10^. Interestingly, in order to improve properties of GNS, it may be modified by using N-doping to generate N-GNS^11–13^. In the case of Pt 10–70 wt.%/GNS, they perform the outstanding properties and Pt Subnano clusters were exist on GNS where those are never be found on the other carbon supporting materials^2,3^. That is caused size and shape of Pt clusters may affect the properties of interaction between Pt clusters and graphene sheets^14^. The other groups reported Mg clusters in 3D hierarchical structure (3DHS) have a superior cycling stability of up to 1350 h^15^. The shape, direction, size and alloy of Pt may affect the ORR catalytic activity and durability^16–19^. The interaction between metal–support may induce charge transfer between metal and support, tame electronic structure of supported metals, impact adsorption energy of reaction intermediates, and eventually change the catalytic performance^20,21^. However, the metal loading density must be kept low (usually below 0.5%) to avoid the formation of metal nanoparticles through sintering^22^. The nanoscales materials may be designed referring to controllable transportation, separation, classification of nano-objects^23^ and type of supporting materials^24^. Their electronic properties and catalytic activity are tuned by the interaction between the central metal and the neighboring surface atoms, and their atomically dispersed nature allows for metal utilization of up to 100%^25^. Graphene as well as a supporting material may assist to homogenize and disperse noble metals^26,27^, to generate metal clusters^28^, reducing CO oxidation reaction by assisting strong interactions between support–cluster^29^, and protecting Pt cores on fuel cells operating system^30^. Pt nanoparticles have been applied as catalysts on fuel cells^31^.

The reducing Pt nanoparticles size tends to increasing electrochemical surface area (ECSA) and decreases loading. Noteby, smaller nanoparticles generally have many weaknesses those are reducing lifetime in electrochemical devices; weak interaction between platinum and carbon black, resulting nanoparticle dissolution; aggregation and detachment, leading to PEFC performance degradation; and carbon corrosion and associated loss of ECSA over time at high cathode potentials^32^. In addition, the small clusters may cause break the scaling relationship in catalysis and may exceed the intrinsic limit on catalytic activity^33^. It means the particle size and morphology may affect CO species bonding on clusters sites^34^.

Commonly, the particle size, shape, chemical composition, metal–support interaction, metal–reactant/solvent interaction and different size (single atoms, nanoclusters, and nanoparticles) of metal exhibits different performance on the catalytic properties of metal catalysts^35^.

In this study, we focus on clarifying the nature of the strong interaction between platinum nanoparticles and GNS. The disorder and non-uniform of Pt clusters are interesting to explore. However, the fabrication of Pt clusters and their size effect on Pt/GNS catalysts (concerning Pt mass loading below 10%) have not been reported yet.

## Results

### TG/DTA

**Figure 1:**
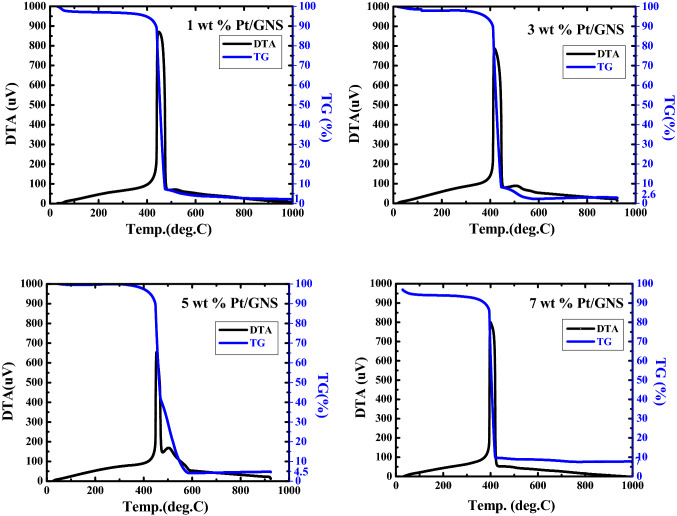
TG/DTA thermograms for 1–7 wt.% Pt/GNS.

**Figure 2:**
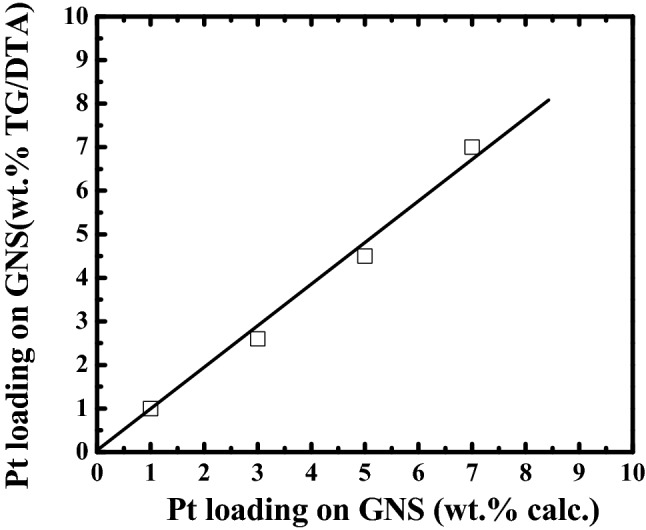
Pt amount on GNS (wt.%) measured by TG/DTA versus calculated Pt amount on GNS (□).

Firstly, we measured amounts of Pt on GNS on 1–7 wt.% Pt/GNS catalysts by using TG/DTA in order to make sure that amount of Pt deposited on GNS. As shown in TG/DTA data (Fig. 1), the amounts of Pt are exactly similar with their calculated as well as the prepared catalysts (Fig. 2). Those data indicate that all the Pt atoms of catalyst precursors exist on Pt/GNS catalysts. Base on data, the amount of Pt on GNS may be controlled exactly with GNS, and then we can evaluate properties of Pt (particle size, electronic structure and catalytic activity) as a function of the Pt amount.

### XRD

**Figure 3:**
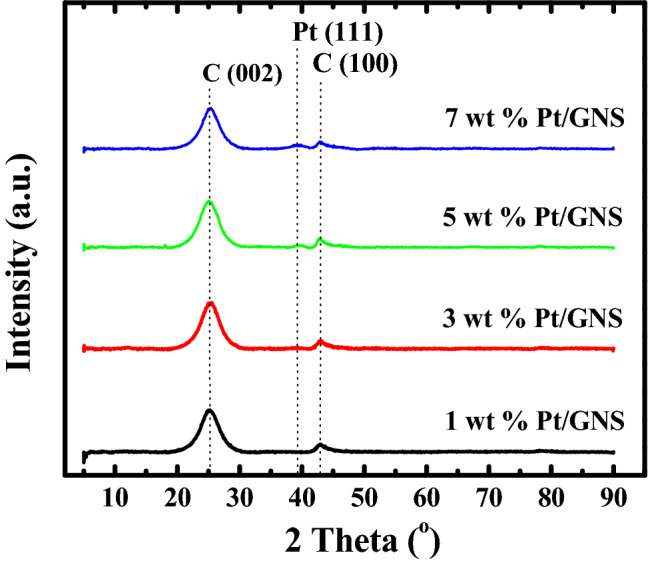
XRD patterns of 1–7 wt.% Pt/GNS.

In order to prove that Pt atoms are well deposited on GNS surfaces, we characterized Pt/GNS catalysts with XRD. The Fig. 3 shows XRD patterns of 1–7 wt.% Pt/GNS. All the patterns gave peaks at 2Ө Bragg angles around 25° and 39° corresponding to C (002) and Pt (111), respectively. The C (002) peak appears weak and broad for Pt/GNS catalysts. Note that the C (002) peak usually appears sharp at 26.5° for graphite and graphite oxide (GO) corresponding to the interlayer distance of *d* = 3.4 Å ^36^ and *d* = 8.4 Å^2^, respectively. The weak and broad peaks at 26.5° (Fig. 3) shows the formations of graphene sheets (stacked graphene) are present due to the peaks of graphite and GO do not appear.

The Pt (111) peaks at 1–7 wt.% Pt/GNS catalysts clearly seem weak and broad. The weak and broad peak of Pt (111) on Pt/GNS catalyst indicates that Pt nanoparticles (probably 1–3 nm) are present on them. Then, the particle sizes of Pt were estimated by TEM.

### TEM

**Figure 4:**
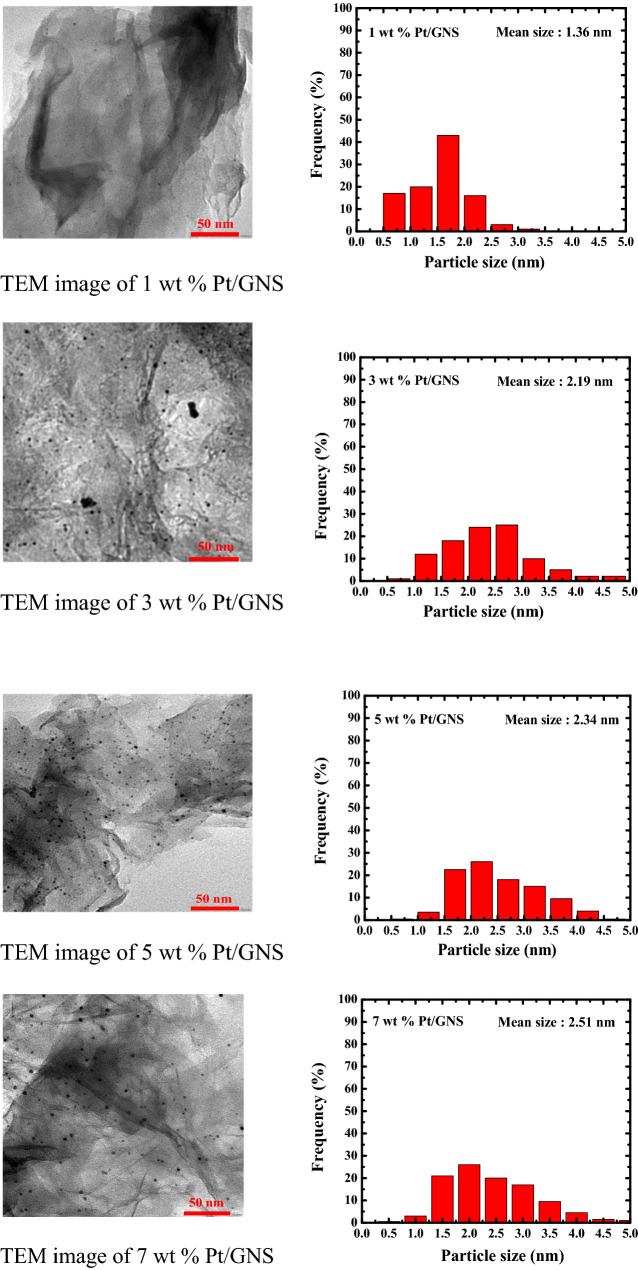
TEM images and histograms of 1–7 wt.% Pt /GNS.

**Figure 5:**
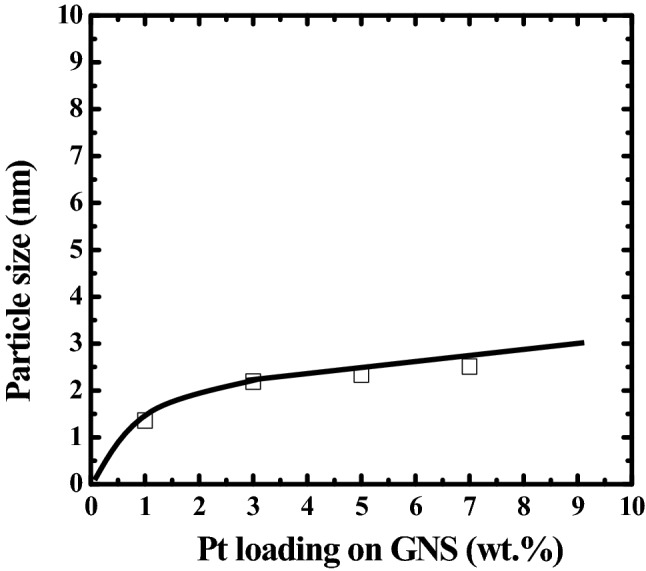
Average Pt particle size (nm) estimated by TEM versus Pt amount on GNS.

The average Pt particle size, its distribution and dispersion as well as the function of Pt loading on Pt/GNS were evaluated by TEM. The TEM images and histograms of Pt particle size distributions which were measured 650 Pt particles size of 1–7 wt.% Pt/GNS may be seen in Fig. 4, respectively. The TEM images clearly show Pt particles are well dispersed and homogenously distributed on GNS without aggregation even after heating treatment in N_2_/H_2_ (4 : 1 v/v) at 400 °C in the catalyst preparation process. The average Pt particles sizes gradually become large with increasing Pt amount on 1–7 wt.% Pt/GNS (1.3–2.5 nm). On the other word, the average particle size of Pt linearly increases with increasing amount of Pt (Fig. 5).

Interestingly, the Pt subnano-clusters (below 1 nm) are seen for 1–7 wt.% Pt/GNS catalysts (around 5–35%). The 1 wt.% Pt/GNS has the most Pt subnano-clusters (35%) among the others and the average Pt particle size is estimated to be 1.36 nm (Fig. 4). The Pt subnano-clusters may be expected to increase catalytic activity leading to high surface area of Pt and high efficiency in the Pt usage for Pt electro catalysts.

### XPS

**Figure 6:**
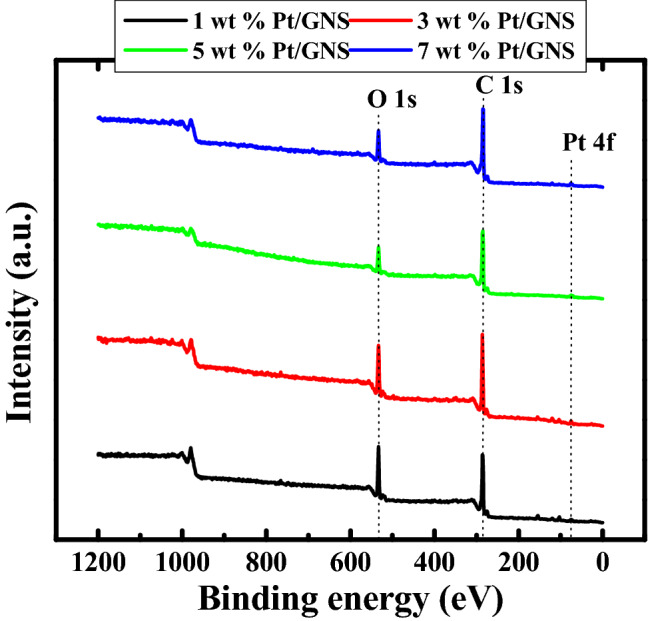
XPS spectra of 1–7 wt.% Pt/GNS (Wide Scans).

Furthermore to elaborate the Pt electronic state of 1–7 wt.% Pt/GNS catalysts, we used XPS. The XPS spectra for 1–7 wt.% Pt/GNS catalysts may be seen in Fig. 6. On the XPS wide scan spectra clearly appear the very weak peaks at 70–74 eV for 1–7 wt.% Pt/GNS, indicating Pt 4f. definitely exist on them. The detail of atomic ratio for 1–7 wt.% Pt/GNS catalysts are shown in Table 1. This data shows that Pt/GNS catalysts consist of Pt, C and O atoms. The atomic ratio of Pt increases with increasing Pt amount on GNS at 1–5 wt.% Pt/GNS and slightly decreases for 7 wt.% Pt/GNS. Importantly, Pt atoms are well deposited on GNS.

**Table 1:** Quantification atomic (%).

Samples	C 1s	O 1s	Pt 4f.
Pt 1 wt.%/GNS	63.5	34.8	1.7
Pt 3 wt.%/GNS	65.4	32.5	2.1
Pt 5 wt.%/GNS	63.6	34.0	2.4
Pt 7 wt.%/GNS	66.8	31.4	1.8

**Figure 7:**
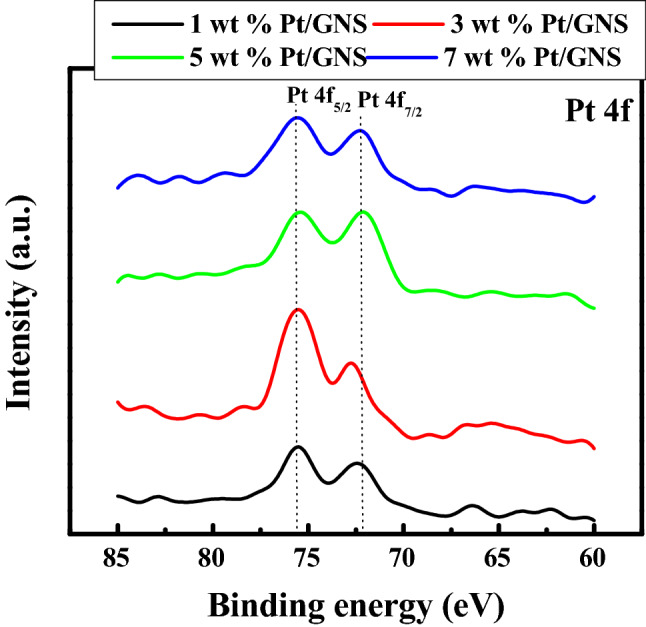
XPS Pt spectra of 1–7 wt.% Pt/GNS (Narrow Scans).

We thus analyzed narrow scan for 1–7 wt.% Pt/GNS catalysts in order to deeply know state of Pt on Pt/GNS catalysts. Figure 7 shows there are doublet peaks at low energy band (72.0–73.0 eV) and high energy band 75.0–76.0 eV, attributed as Pt 4f_7/2_ and Pt 4f_5/2_, respectively for 1–7 wt.% Pt/GNS. These peaks (Pt/GNS) are totally different with Pt/CB, indicating the Pt chemical state may be modified by GNS^3^. As shown in Fig. 7, the Pt 4f_7/2_ binding energy is 72.1 and 72.3 eV for 5 and 7 wt.% Pt/GNS, respectively, indicating that Pt binding energies are totally different with the bulk platinum metal, PtCl_2_ and PtCl_4_ (as a reference Pt 4f_7/2_ (71.2 eV bulky Pt metal), (73.4–73.8 eV) and (75.3–75.1 eV))^37^. It means that the Pt states of 5 and 7 wt.% Pt/GNS catalysts are metal, probably Pt nano particles. In contrast, Pt binding energies of 1 and 3 wt.% Pt/GNS are 72.4 and 72.8 eV, respectively. This data is closely to Pt(OH)_2_ and Pt-Oxides, regarding to reference 72.4–72.8 and 73.8–75 eV, respectively^37^. They indicate that the shifting Pt 4f_7/2_ binding energies are affected by Pt particle sizes. The small and weak Pt 4f. peaks indicate that Pt subnano-clusters are formed on those catalysts.

In the case of 1 and 3 wt.% Pt/GNS catalyst (72.4 and 72.8 eV), the Pt 4f_7/2_ binding energy is shift slightly to higher than 5 and 7 wt.% Pt/GNS catalyst (72.1 and 72 eV). It indicates that the large Pt particle size exist on 5 and 7 wt.% Pt/GNS catalysts.

Based on the XPS data, they show that the Pt binding energy decreases with increasing Pt particle size. The Pt 4f. line shifts towards higher binding energy for decreasing Pt particle size and Pt 4f. levels move toward lower binding energy for increasing Pt particle size. The small and weak Pt 4f. peaks and shift to positive binding energy are possible contributed by strong interaction between Pt and GNS or Pt particle sizes effects^38^. However, the large Pt particle size causes the Pt 4f. binding energy shifts to negative binding energy. It generates the weak interaction between Pt and GNS. There are three factors can contribute to the core level binding energy shift: initial state effects associated to changes in the local electronic structure (valence electron configuration), final state effects due to changes in the relaxation process (extra-atomic response to the positively charged photo hole), and cluster charging^39^.

### CO tolerance

**Figure 8:**
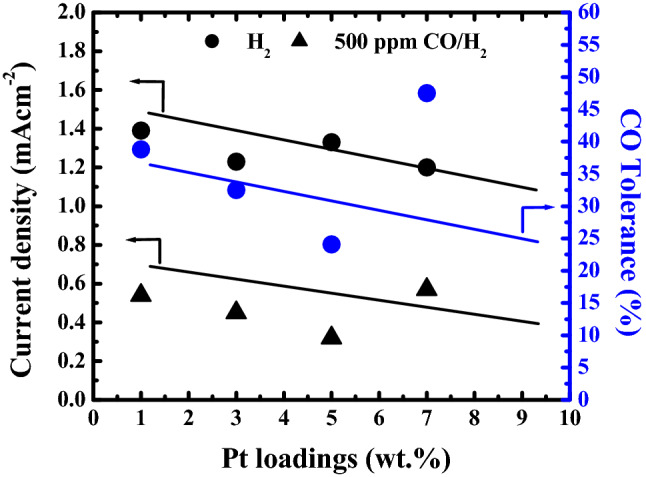
Graph current density and CO tolerance vs Pt loading on GNS (wt.%) (CV measurement).

The electro catalytic activities for 1–7 wt.% Pt/GNS catalysts were evaluated by measuring CO tolerance. The CO tolerance was evaluated in HOR by CV measurement in 0.1 mol dm^−3^ HClO_4_ at 60^o^ C, and scan rate 10 mVs^−1^ for 1 h in pure H_2_ and in H_2_ with 500 ppm CO. The polarization curves of the HOR for 1–7 wt.% Pt/GNS (Fig. 8). As shown in Fig. 8, in the presence of pure H_2_ and H_2_ with 500 ppm CO, the catalytic activities for all the catalysts significant are be affected by Pt amount on Pt/GNS catalysts.

The CO tolerance for 1, 3, 5 and 7 wt.% Pt/GNS catalysts remained at *ca.* 39, 33, 25, and 48% (1.4, 1,3, 1.45, and 1.2 mAcm^−2^) of that in pure H_2_ at—0.1 V versus Ag/AgCl, respectively. It indicates that the 7 wt.% Pt/GNS catalyst has the highest electro catalytic activity among the others. This is caused the Pt size is larger on 7 wt.% Pt/GNS catalyst (particle size effect).

In the case of 1 wt.% Pt/GNS, its HOR activity remained at *ca.* 39% (1.4 mAcm^−2^) of that in pure H_2_ at—0.1 V versus Ag/AgCl. It indicates that this catalyst has low CO tolerant. It is caused many Pt subnano-clusters were formed on it.

Figure 8 shows clearly, the CO tolerance of 7 wt.% Pt/GNS catalysts dramatically drop being 9% (0.56 mAcm^−2^) of that in pure H_2_ at—0.1 V versus Ag/AgCl for 1 wt.% Pt/GNS catalyst. This is possible because decreasing the amount Pt subnano-clusters were formed. It causes Pt particle size become small. This data indicates that electro catalytic activity of 1 wt.% Pt/GNS catalyst is less than 7 wt.% Pt/GNS catalyst due to Pt particle size effect.

### ORR

**Figure 9:**
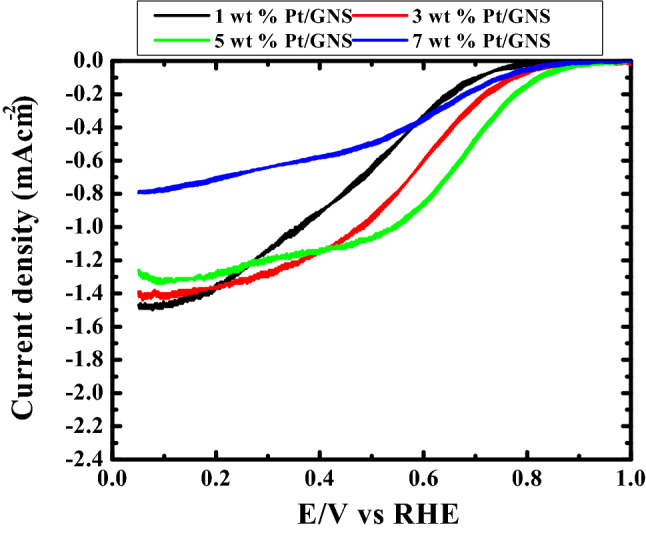
Cyclic voltammograms for the ORR using a Pt 1–7 wt.% Pt/GNS catalysts in the absence of O_2_ or under conditions of O_2_ saturation; Rotating ring-disk voltammogram (500 rpm) 0.1 mol dm^−3^ HClO_4_, scan rate 10 mVs^−1^ of the ORR.

Figure 9 show the ORR voltammograms and threshold voltages (onset potensial) for 1–7 wt.% Pt/GNS catalysts, respectively. The position and the shape of the stripping peak are extremely sensitive to the Pt particle size^40^.

As shown in Fig. 9, the ORR peaks potential for 1, 3, 5 and 7 wt.% Pt/GNS catalysts appear at 0.8066, 0.8278, 0.9511 and 0.9102 V versus Ag/AgCl. These data indicate that Pt particle size affect ORR catalytic activity for Pt/GNS catalysts.

In the case of 1–5 wt.% Pt/GNS catalysts, the onset potential peaks gradually increase tends to increasing Pt amount on Pt/GNS, and the 7 wt.% Pt/GNS little beat decreases comparing to 5 wt.% Pt/GNS (Fig. 9). This data indicates that the Pt particle sizes each of them are different. That is possible due to 1 and 3 wt.% Pt/GNS catalysts are composed much more Pt subnano-clusters than 5 and 7 wt.% Pt/GNS catalyst. It indicates that the Pt particle size of 5 and 7 wt.% Pt/GNS is larger than 1 and 3 wt.% Pt/GNS catalysts. It means the adsorbed O_2_ on 5 wt.% Pt/GNS is easier oxidized compare to 1, and 3 wt.% Pt/GNS catalysts. This is caused Pt subnano-clusters were formed on it.

For 7 wt.% Pt/GNS catalyst, the O_2_ oxidation is easier than 1 and 3 wt.% Pt/GNS catalysts. This is possible due to the large Pt particle size exist on this catalyst. The large Pt particle size contributes to the O_2_ oxidation potential peak and also threshold peak shifter to low potential peak compares to the others catalyst. It indicates that adsorbed O_2_ is more difficult oxidized for the large Pt particle size. This is possible due to the Pt particle size effect.

Figure 9 shows that the starting potential for CO oxidation increases with increasing Pt amount on GNS. It means the Pt particle size affect the CO oxidation on the catalysts.

### Mass activity

**Figure 10:**
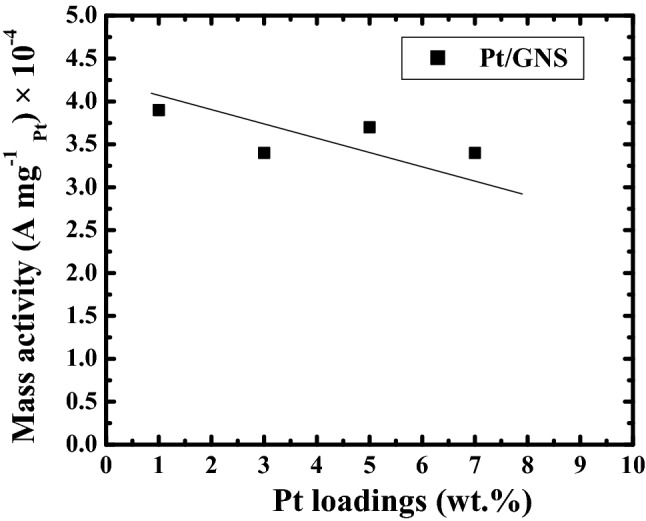
Graph mass activity vs Pt loading on GNS (wt.%) (CV measurement).

The mass activity of 1–7 wt.% Pt/GNS catalysts was evaluated base on CO-tolerance measurements. Those data may be expected to know efficiency usage and catalytic acivity of Pt/GNS catalysts related to Pt loadings on GNS. The mass activity of Pt/GNS at several of Pt loading on GNS may be seen in Fig. 10. Note that the Pt particle sizes for 1–7 wt.% Pt/GNS are 1.36, 2.19, 2.34 and 2.51 nm, respectively. It clearly shows that the mass activity numbers for 1–7 wt.% Pt/GNS decrease with increasing Pt loading on GNS. It means that the Pt particle sizes may affect the Pt mass activity. The high mass activity for 1 wt.% Pt/GNS is caused there are many Pt subnano-clusters exist on it.

### Electrochemically active surface area (ECSA)

The ECSA is calculated from the coulombic charge (Q) from H^+^ ion adsorption–desorption (−0.2–0.1 E (V) vs Ag/AgCl area). It may be evaluated by using CV measurement. The calculation formula for ECSA_H_ is^3^:

$$ {\text{ECSA}}_{{\text{H}}} = \frac{{Q \left( {\mu C/cm^{2} } \right)}}{{210 \left( {\mu C/cm^{2} } \right)}} \times \frac{1}{{Pt \left( {\mu C/cm^{2} } \right)}} $$

**Figure 11:**
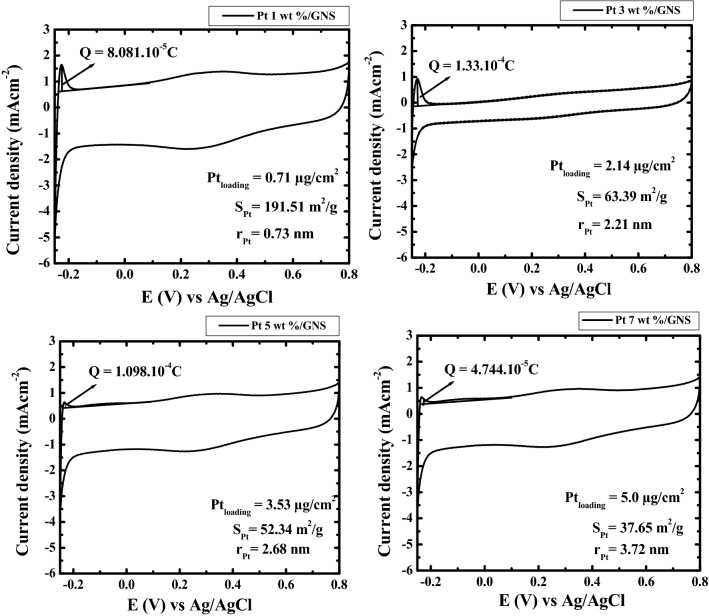
Polarization curves of ECSA_H_ for 1–7 wt.% Pt/GNS on a rotating disk electrode (RDE) measured in 0.1 mol dm^−3^ HClO_4_ at 60 °C, where H_2_ gas is bubbled. Rotating speed 500 rpm and scan rate 10mVs^−1^ between—0.20 and 0.1 vs Ag/AgCl.

The ECSA data of 1–7 wt.% Pt/GNS may be seen in Fig. 11. The Pt particle sizes linearly increase with increasing Pt loadings on GNS. Those data are also consistent with the surface area for 1–7 wt.% Pt/GNS. The ECSA_H_ results may prove that the Pt particle sizes dependence to Pt loadings on GNS.

### CO-stripping

**Figure 12:**
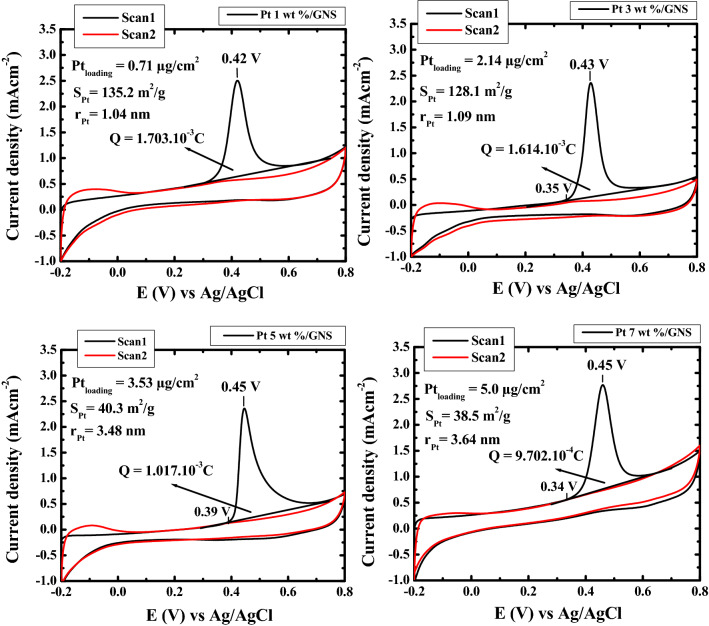
CO stripping voltammogram of 1–7 wt.% Pt/GNS, respectively on a rotating disk electrode (RDE) measured in 0.1 mol dm^−3^ HClO_4_ at 60 °C. Rotating speed 500 rpm and scan rate of 10 mVs^−1^ between—0.20 and 0.8 vs Ag/AgCl.

The electro-oxidation of CO adsorption on Pt surface may be represented by CO stripping. It was measured with CV measurement too. The peak charge Q_CO_ was used to calculate Pt surface area at 0.3–0.6 E (V) vs Ag/AgCl area (Fig. 12). The calculation formula for ECSA_CO_ is shown below as^3^:

$$ {\text{ECSA}}_{{{\text{CO}}}} = \frac{{Q \left( {\mu C/cm^{2} } \right)}}{{420 \left( {\mu C/cm^{2} } \right)}} \times \frac{1}{{Pt \left( {\mu C/cm^{2} } \right)}} $$

Figure 12 shows increasing Pt loadings on GNS cause surface area, electro-oxidation and igniton voltage for 1–7 wt.% Pt/GNS increase. That is possible due to effect of particle sizes of Pt. Therefore, the catalytic activity of Pt/GNS is significantly different due to particle size effect.

## Discussion

### Subnano-clusters

TEM data indicates that many Pt subnano-clusters were formed for 1 wt.% Pt/GNS. This data is also consistent with the ECSA_H_ data and having the Pt largest surface area (S_Pt_ = 191. 51 m^2^/g) among the others. However, Pt subnano-clusters coexist with Pt nanoparticles for 3–7 wt.% Pt/GNS. It causes the average particle size of Pt and S_Pt_ will be larger and smaller than 1 wt.% Pt/GNS (ECSA_H_ and ECSA_CO_). The Pt subnano-clusters were formed on GNS may be expected for preparing a highly catalytic electrocatalyst. This is possible due to Pt subnano-clusters have large surface area. Thus, the interparticle distances on Pt particles are sufficient leads to increase catalytic activity^41^.

X-ray diffraction (XRD) patterns for 1–7 wt.% Pt/GNS catalysts are weak and broad, indicating Pt subnano clusters are formed.

XPS data indicate that Pt subnano-clusters have strong interaction between Pt and GNS. It may contribute to forming a stable Pt-cluster graphene interface^7^. This is very important due to the strong interaction between Pt and graphene may induce some modulation in the electronic structure of the Pt clusters^42^.

### Catalytic activity

The HOR catalytic activity of 7 wt.% Pt/GNS is higher among 1, 3 and 5 wt.% Pt/GNS catalysts and its ORR catalytic activity is higher than 1 and 3 wt.% Pt/GNS catalyst. It is caused adsorbed CO on 7 wt.% Pt/GNS is more readily oxidized than 1, 3 and 5 wt.% Pt/GNS catalysts. It indicates that graphene may be expected to improve electrocatalytic activity. The Pt subnano-clusters show more resistant to CO. This is possible due to the distance between Pt atom and Pt atom is shorter. The shorter distance can assist to reduce energy barrier of CO_2_ formation. Thus, CO_2_ formation can be easily occur for Pt subnano-clusters. It indicates the catalytic activity of Pt subnano-clusters is high.

Based on HOR measurement, the CO-tolerance of Pt/GNS catalysts tend to increase with increasing Pt particle size. It means Pt particle size affects CO-tolerance value of Pt/GNS catalysts. This is possible due to the number of active surface sites catalyst becomes larger with increase of Pt particle size on GNS. The 7 w t% Pt/GNS catalyst shows a very high CO tolerance. It means even the amount of Pt subnano-clusters on GNS is very small. it may improve electrocatalytic activity of catalyst. It is caused Pt subnano-cluster may serve many sites for strong interaction between Pt and GNS. Therefore, Pt subnano-clusters may be expected to be a good catalyst especially for anode in PEFCs due to it has superior CO tolerance.

The significant difference in the CO tolerance depending on Pt particle sizes suggests the modification of the Pt electronic structure by the interface interaction between Pt and graphene. In the previous surface science study using a Pt-deposited graphite model catalyst, we have found that a significant chemical interaction exists at the interface between the graphite surface and the Pt catalyst particles leading to a modification in the electronic structure of the Pt catalysts^43^. It is thus suggested in this study that Pt clusters with smaller sizes interact more significantly with graphene via the same type of π–d hybridization^44^. In the first principles calculation Pt nanoparticle on GNS has been expected to be more resistant to the CO poisoning^45^. Thereby, Pt subnano-clusters have high catalytic activity. This is a good new due to we can reduce Pt usage and improve Pt utilization by using Pt subnano-clusters were formed on GNS.

The catalytic activity may be also characterized by mass activity measurement. The high mass activity of electrocatalyst can contribute to increase the catalystic activity of electrocatalyst. Base on the mass activity data (Fig. 10). Interestingly, the Pt 1 wt.%/GNS has the highest mass activity among the others even the Pt loading is very small. It occurs due to the existence of Pt subnano-clusters (~ 35%) is much more comparing to Pt 3–7 wt.%/GNS (~ 15, 5 and 2.5%, respectively) (TEM data). It means the Pt particle size may affect the catalytic activity of Pt (particle size effect). Thereby, Pt subnano-clusters can be expected to reduce Pt usage, because the small Pt loading on GNS is still being able to generate high electrocatalytic activity.

The CO stripping data (Fig. 12) show the ignition and electro-oxidation voltage on 1 wt.% Pt/GNS is lowest among the others, suggesting it has higher catalytic activity than 3–7 wt.% Pt/GNS for CO oxidation. At 1 wt.% Pt/GNS, threshold voltage is very low as 0.31 V, indicating a superior catalytic activity of Pr subnano-clusters on GNS. Meanwhile, the threshold voltages shift to higher voltage with increasing Pt loading meaning catalytic activity is low for large particle size (particle size effect).

## Methods

GNS were prepared by modified Hummers method. The various of Pt amounts on GNS were prepared with impregnation method^2,3^. The 1–7 wt.% Pt catalysts were deposited onto GNS using a Pt precursor of [H_2_PtCl_6_^**.**^6H_2_O] (Alfa Aesar, A Johnson Matthey Company), respectively. Briefly, the ethanol solution of the Pt precursor was mixed with the ethanol solution of GNS and was stirred for 6 h. Then, the product was collected by filtration and dried in air at 100 °C for 24 h. Finally, the product was subjected to heat treatment in a hydrogen (H_2_) (25 mL/min) stream at 400 °C for 2 h in a furnace. The products were collected and attributed as 1–7 wt% Pt/GNS catalysts, respectively. Then, the 1–7 wt.% Pt/GNS catalysts were characterized by Thermogravimetric/Differential thermal analysis (TG/DTA), X-ray diffraction (XRD), Transmission electron microscope (TEM), X-ray photoelectron spectroscopy (XPS) and electrochemical measurement. TG/DTA measurements were carried out using TG/DTA6300, Seiko Instruments Inc. (Reference: Pt-pan; Gas/flow speed: Air/200 mL/min; temperature measurement: 50–1000 °C; Rate: 10 °C/min, respectively). XRD measurements were performed at room temperature employing a two circle diffractomer (PANalytical PW 3050 Philips X`Pert Pro, Cu—K_α_ radiation of 1.541 Å, without monochromator), installed at a line focus X-ray generator. A reflection free Si plate was used as a sample stage. CuK_α_ radiation obtained by reflection from a singly bent HOPG crystal was used. Diffraction pattern was recorded using a solid state detector (PANalytical X`Celerator) with a scan speed of 0.005 deg. (in 2Ө)/sec up to 90 degrees. TEM images were recorded using JEOL JEM-1400 Jeol Datum LTD, Japan, electron microscope operated at 80 kV, resolution lattice image 0.20 nm and resolution point image 0.38 nm. XPS measurements were carried out using JEOL, JPS 9010 TR (X-ray source Al-Kα, 1486.6 eV, pass energy 50 eV, energy resolution 1.88 eV which was calibrated using Ag 3d_5/2_ by measuring a clean Ag sample, the uncertainty of binding energy ± 0.05 eV). The electrochemical measurements were examined using PGSTAT PG12, AUTOLAB Potentiostat/Galvanostat).

The catalytic activity of 1–7 wt.% Pt/GNS catalysts were tested for HOR. The HOR electrocatalytic activity for Pt 1–7 wt.%/GNS catalysts, respectively, was tested by using a rotating disk electrode (RDE) equipped with in a three electrodes system. Briefly, 1 mg of 1–7 wt.% Pt/GNS catalysts, respectively, were ultrasonically suspended in 5000 μL of nafion-methanol mixture (1:50 ^v^/_v_) solution (5 wt.% nafion Aldrich solutions) to prepare catalyst ink solution. Then, Pt loadings (0.02, 0.06, 0.10, 0.14 μg) for 1, 3, 5, and 7 wt.% Pt/GNS catalysts, respectively, were transferred with an injector to clean a glassy carbon disk electrode (6 mm diameter, with area of 0.28 cm^2^) (the thickness of nafion film is 0.1505 μm) and dried at room temperature for 30 min. The measurements were done using a rotating disk electrode (RDE) with three-compartment cell. The working electrode compartment was separated from other two compartments by fritted glass discs. The working electrode was a glassy carbon disk. The counter electrode was a platinum wire (0.25 mm diameter). The reference electrode was a silver/silver chloride electrode (Ag/AgCl). All measurements were performed in 0.1 M HClO_4(aq)_ at 60 °C under a flow of pure H_2_ or in a mixture of 500 ppm CO/H_2_ for 1 h and the RDE speed is 500 rpm. Polarization curves were recorded at the scan rate of 10 mV s^−1^, and the current density at—0.1 V vs Ag/AgCl was sampled for evaluation of HOR current density.

The electrochemical active surface area (ECSA) for 1–7 wt.% Pt/GNS catalysts were examined by using hydrogen adsorption/desorption and CO stripping measurements, respectively. Commonly, hydrogen adsorption/desorption in an electrochemical is carried out to evaluate ECSA^36^. For ECSA_H_ measurements, the Pt loading (0.2, 0.6, 1.0, 1.4 μg) for 1–7 wt.% Pt/GNS catalysts, respectively, were loaded onto a glassy carbon disk electrode (0.28 cm^2^) with diluted (1:50 in methanol) 5 wt% Nafion solutions (Aldrich), respectively. The measurements were done in a three electrodes system in 0.1 M HClO_4_ at 60 °C, with Pt-wire and Ag/AgCl as the counter electrode and reference electrode, respectively. The working electrode was the catalyst supported glassy carbon disk. The cyclic voltammograms (CVs) were obtained at—0.25 to 0.8 V versus Ag/AgCl with scan rate of 10 mVs^−1^ in a nitrogen saturated.

For CO-stripping measurements, the Pt loading (0.2, 0.6, 1.0, 1.4 μg) for 1–7 wt.% Pt/GNS catalysts, respectively, were loaded onto a glassy carbon disk electrode (0.28 cm^2^) with diluted (1:50 in methanol) 5 wt% Nafion solutions (Aldrich), respectively. CO was adsorbed to the surface of the working electrode by bubbling 3% CO/H_2_ into the electrolyte solution of 0.1 M HClO_4_ at 60^o^ C for 1 h, while holding the working electrode potential at—0.15 V vs. Ag/AgCl. After 3% CO/H_2_ bubbling, the gas was switched to nitrogen for 30 min and the potential was scanned from—0.2 to 0.8 V vs Ag/AgCl to record the CO-stripping voltammogram^46^.

## Conclusions

We have studied the properties of 1–7 wt.% Pt/GNS. The Pt subnano-clusters were formed at the 1–7 wt.% Pt/GNS catalysts. 7 wt.% Pt/GNS catalyst exhibits the best performance with Pt particle size (2.51 nm, superior CO tolerance (48%) compared to the CO-free H_2_ under 500 ppm CO level), and strong interaction between Pt and graphene. Therefore, Pt subnanoclusters can be expected for reducing Pt usage and improving Pt utility. Note that, the Pt particle sizes effect depend on the amount of Pt subnano-clusters on them.

## Supplementary Information

Supplementary Information.
