# Supplementary material for: The loading effect of Pt clusters on Pt/graphene nano sheets catalysts
Source: Sci Rep. 2021 Jan 28;11:2532. doi: 10.1038/s41598-020-80472-1 (PMC7844224; doi:10.1038/s41598-020-80472-1)
Supplement: Supplementary file 1 — Supplementary Information. [file 41598_2020_80472_MOESM1_ESM.docx]

**The Loading Effect of Pt clusters on Pt/Graphene Nano Sheets Catalysts**

*Rikson Siburian^1,2*^, Ab M. M Ali^3*^, Kerista Sebayang^2,3^, Minto Supeno^1,2^, Kerista Tarigan^2,4^, Crystina Simanjuntak^1^, Sri Pratiwi Aritonang^1^, Fajar Hutagalung^1^*

^1^Chemistry Department, Faculty of Mathematics and Natural Sciences, Universitas Sumatera Utara, Padang Bulan, Medan, Indonesia (20155)

^2^Carbon Research Center, Universitas Sumatera Utara, Padang Bulan, Medan, Indonesia (20155)

^3^Faculty of Applied Sciences, Universiti Teknologi MARA, Shah Alam, Selangor, Malaysia (40450)

^4^Department of Physics, Faculty of Mathematics and Natural Sciences, Universitas Sumatera Utara, Medan, Indonesia (20155)

*Corresponding email: [rikson@usu.ac.id](mailto:rikson@usu.ac.id); ammali@uitm.edu.my.

SUPPORTING INFORMATION

Figure 1S. XPS C spectra of 1–7 wt % Pt/GNS (Narrow Scans)

Figure 2S. XPS O Spectra of 1–7 wt % Pt/GNS (Narrow Scans)

Figure 1S. XPS C spectra of 1–7 wt % Pt/GNS (Narrow Scans)

Figure 2S. XPS O spectra of 1–7 wt % Pt /GNS (Narrow Scans)
